# Targeting the mTOR Signaling Pathway Through miR‐100 and miR‐101 in De Novo Acute Myeloid Leukemia: Implications for Therapeutic Intervention

**DOI:** 10.1002/cnr2.70264

**Published:** 2025-08-03

**Authors:** Maryam Kargar, Mehdi Allahbakhshian Farsani, Javad Garavand, Mahnaz Gorji, Mohammad Rafiee, Seyed Sobhan Bahreiny, Mohammad Hossein Mohammadi

**Affiliations:** ^1^ Department of Laboratory Hematology and Blood Bank, School of Allied Medical Science Shahid Beheshti University of Medical Sciences Tehran Iran; ^2^ HSCT Research Center, Laboratory Hematology and Blood Banking Department, School of Allied Medical Sciences Shahid Beheshti University of Medical Sciences Tehran Iran; ^3^ Department of Laboratory Sciences, School of Allied Medical Sciences Ahvaz Jundishapur University of Medical Sciences Ahvaz Iran; ^4^ Department of Medical Laboratory Sciences, School of Para Medicine Hamadan University of Medical Sciences Hamadan Iran; ^5^ Medical Basic Sciences Research Institute, Physiology Research Center, Department of Physiology, School of Medicine Ahvaz Jundishapur University of Medical Sciences Ahvaz Iran

**Keywords:** acute myeloid leukemia, microRNA‐100, microRNA‐101, *mTOR/AKT/PI3K* pathway

## Abstract

**Background:**

Microribonucleic acid (MicroRNAs/miRNAs) play a significant role in cancer progression by changing cellular functions through the modulation of protein expressions. The potential of different miRNAs to alter the expression of the mammalian target of rapamycin (*mTOR*)/protein kinase B (*PKB* or *AKT*)/phosphatidylinositol‐3‐kinase (*PI3K*) signaling cascade, a key pathway in the progression of acute myeloid leukemia (AML), has been demonstrated across different types of cancers.

**Aims:**

This study aims to explore the effects of *miR‐100* and *miR‐101* on the *mTOR/AKT/PI3K* signaling pathway in AML.

**Methods and Results:**

Initially, we employed the TargetScan, miRDB, and miRanda databases to identify the target proteins of *miR‐100* and *miR‐101*. Following a comprehensive analysis, we identified the *mTOR*/*AKT*/*PI3K* signaling pathway as a significant target for investigation in patients with AML. In this case–control study, the expression levels of miRNAs and genes were analyzed in 21 AML patients and 9 healthy controls using quantitative reverse transcription polymerase chain reaction (qRT‐PCR). The results showed that *miR‐100* was significantly upregulated, while *miR‐101*, *mTOR*, and *PI3K* were downregulated in AML patients. Correlation analysis revealed a negative relationship for *miR‐100* and a positive one for *miR‐101* with *mTOR*, but no significant correlation with *AKT1* and *PI3K* genes.

**Conclusion:**

These findings suggest that both *miR‐100* and *miR‐101* act as tumor suppressors via the *mTOR/AKT/PI3K* signaling pathway, highlighting their potential as therapeutic targets in AML.

**Abbreviations**
AML: Acute myeloid leukemia
FDA: Food and drug administration
microRNA, miRNA: Micro ribonucleic acid
*PI3K*
: Phosphatidylinositol‐3‐kinase
*mTOR*
: Mammalian target of rapamycin
ALL: Acute lymphoblastic leukemia
T‐ALL: T‐cell acute lymphoblastic leukemia
AMPK: AMP‐activated protein kinase
CMV: Cytomegalovirus
UTR: 3′‐untranslated region

## 1 Introduction

Acute myeloid leukemia (AML) is the most prevalent type of acute leukemia in adults, characterized by differentiation arrest and malignant expansion of clonal myeloid progenitor cells. It can be classified into various subtypes based on the cell type and grade of maturity [1]. Although new drugs for affected individuals have been approved in recent years, the 5‐year survival rate remains low, and only about 40% of AML patients survive in the long term, as most patients experience treatment resistance and relapse [1, 2]. Therefore, a deeper understanding of the molecular mechanisms of leukemogenesis and the exploration of new therapeutic approaches are essential to improve outcomes.

Microribonucleic acids (MicroRNAs/miRNAs) are a class of small noncoding RNA molecules consisting of 17–23 nucleotides [3]. Alterations in miRNA‐mediated posttranscriptional regulation alongside genetic and genomic abnormalities have been shown to play a crucial role in hematopoiesis and AML [4]. In the context of cancer, miRNAs can act as oncogenes or tumor suppressors and are implicated in treatment outcomes and drug resistance [2].

It has been demonstrated that in pediatric AML, increased levels of *miR‐100* lead to the inhibition of the ataxia‐telangiectasia mutated (*ATM*), thereby preventing apoptosis. This suppression is closely linked to unfavorable prognoses, manifesting as shorter durations of relapse‐free survival and reduced overall survival rates [5, 6]. Conversely, *miR‐101* acts as a tumor suppressor, inhibiting cell proliferation, apoptosis, and metastasis, and is significantly underexpressed in various malignancies [7]. *MiR‐101* contributes to the upregulation of *p21/Cdkn1a* expression. In mixed lineage leukemia (*MLL*)‐rearranged AML, downregulation of *miR‐101* leads to decreased *p21/Cdkn1a* expression, facilitating AML proliferation [8]. These findings suggest that *miR‐100* and *miR‐101* play major roles in AML pathophysiology. Further investigations are required to elucidate the molecular network of miR‐100 and miR‐101 and their downstream targets.

One of the most frequently dysregulated signaling pathways in human malignancies, particularly in AML, is the *mTOR*/*AKT*/*PI3K* pathway [9]. This network is critical for metastasis, invasion, and poor prognosis in various cancers [10]. The role of miRNAs in regulating this cascade has been investigated in various cancers [11]. For instance, *miR‐100* targets *mTOR* in bladder cancer, inhibiting cell growth and arresting the G1 phase without inducing apoptosis [12]. Additionally, *miR‐101* has been shown to reduce cell proliferation and invasion, and enhance apoptosis in endometrial cancer by modulating the *mTOR*/*AKT*/*PI3K* signaling pathway [13].

However, the relationship between *miR‐100*/*miR‐101* and *mTOR*/*AKT*/*PI3K* expression in AML remains elusive. The current research investigates the correlation of *miR‐100* and *miR‐101* with the *mTOR*/*AKT*/*PI3K* pathway to lay the groundwork for future studies of AML pathways, with the hope of enhancing our understanding of AML pathogenesis.

## 2 Materials and Methods

### 2.1 Patients and Samples

Between May 2018 and October 2019, bone marrow (BM) and peripheral blood (PB) specimens were collected from 21 AML patients and 9 healthy controls at Taleghani Hospital. Prior to BM and PB aspiration, none of the participants had undergone chemotherapy, radiation, targeted therapy/immunotherapy, or hematopoietic stem cell transplantation. According to the French–American–British (FAB) classification, 19 patients had non‐M3 AML, and two had AML‐M3. The control group consisted of nine healthy individuals (25–45 years old, median age 31; three men and six women) with normal PB smears, and no prior history of infection or cancer. This study was approved by Shahid Beheshti University of Medical Sciences Ethics Committee (Ethics ID: IR.SBMU.RETECH.REC.1400.693) and was conducted in accordance with the guidelines set by the Taleghani Hospital Ethics Committee, which is a member hospital of Shahid Beheshti University of Medical Sciences. All participants or their guardians gave written informed consent to conduct the biological studies.

### 2.2 Inclusion and Exclusion

For this study, patients diagnosed with AML aged 16 years and older who have not previously received treatment for AML were selected. Patients must provide informed consent to participate in the research. Those with other types of leukemia or hematologic disorders, or with severe comorbid conditions that could interfere with the study, were excluded. Additionally, patients who have previously undergone chemotherapy or radiotherapy for AML were not included in this study.

### 2.3 RNA Extraction

Peripheral blood mononuclear cells (PBMCs) were separated from the BM and PB specimens using Ficoll–Paque Plus (GE Healthcare, Chicago, IL, USA) according to the manufacturer's protocol. Total RNA, including miRNA and mRNA, was extracted using Trizol reagent (Invitrogen, Carlsbad, CA, USA) according to the manufacturer's instructions, the purity of intact RNA was assessed using the Nanodrop 2000 spectrophotometer (Thermo Fisher Scientific Inc., Waltham, MA, USA) and agarose gel electrophoresis was employed to evaluate the total RNA integrity. Only samples displaying two distinct ribosomal RNA bands (28S and 18S) were selected for reverse transcription.

### 2.4 RT‐qPCR

miRNA and mRNA assays with specific primers to hsa‐miR‐100‐5p and hsa‐miR‐101‐3p and *mTOR/AKT/PI3K* were used in quantitative RT‐PCR to determine the relative expression levels of the related mature miRNA and mRNA. Fermentas RevertAidTM Premium First‐Strand cDNA Synthesis Kit (Thermo Fisher Scientific) provided a complete system to efficiently synthesize cDNA from extracted total RNA by reverse transcription. Extracted miRNA was converted into complementary DNA (cDNA) sequences using a universal stem‐loop primer (USLP, Table 2) and RT primer, as previously defined [14]. In summary, 2 μL of RNA was reverse transcribed into cDNA in a 20‐μL reaction for 10 min at 25°C and 60 min at 42°C using 1‐μL random hexamer, 1 μL of RNase inhibitor, 1‐μL dNTP (10 mM), 1‐μL RiboLock RNase Inhibitor (20 U/μL), 2‐μL RevertAid M‐MuLV RT (200 U/μL), 2‐μL USLP, and 4 μL of 5 × reaction buffer according to the manufacturer's instructions. Two selected miRNAs (*miR‐100* and *miR‐101*) and three mRNAs (*mTOR*, *AKT*, and *PI3K*) (Tables 2 and 3) expression levels were quantified using a real‐time quantitative polymerase chain reaction (RT‐qPCR) based on SYBR‐green relative quantification assay. The amounts of miRNAs and mRNAs were calculated relative to the amount of *miR‐16* and *ABL* gene (used as internal controls, respectively) in the same sample, with *miR‐16* serving as a housekeeping gene due to its stable expression across different cell types and conditions, ensuring reliable normalization of miRNA expression levels [15].

The primer mixture was designed to generate sufficient qPCR data with the Rotor‐Gene 6000 using the following PCR program (to study mRNA expression): Stage 1: predenaturation: 95°C for 10 min; Stage 2: followed by 40 cycles of denaturation: 95°C for 10 s, annealing: according to Table 3 for 15 s, extension: 72°C for 15 s; Stage 3: melting curve: 95°C for 10 s, 56.5°C for 15 s, 95°C for 15 s. Correspondingly, the expression of *miR‐100* and *miR‐101* was checked according to Table 1. The PCR products confirmed the amplification and the efficacy of the qPCR technique by melting curve analysis (Figure S1).

**TABLE 1:** Profile PCR schedule for miRNA.

Primer name	Cycle program
miR‐100 (5P)	Stage 1: Predenaturation: 95°C for 10 min; Stage 2: 40 cycles of denaturation: 95°C for 10 s, annealing: 56°C for 10 s, extension: 72°C for 10 s.
miR‐101 (3P)	Stage 1: Predenaturation: 95°C for 10 min; Stage 2: 43 cycles of denaturation: 95°C for 10 s, annealing: 53.6°C for 25 s, extension: 72°C for 15 s.
miR‐16 (5P)	Stage 1: Predenaturation: 95°C for 15 min; Stage 2: 40 cycles of denaturation: 95°C for 13 s, annealing: 57°C for 22 s, extension: 72°C for 20 s.

**TABLE 2:** Primer sequence of microRNA expression analysis and stem‐loop primer for cDNA synthesis.

Primer name	Primer sequences (5′‐3′)	Annealing temp (°C)
miR‐100 (5P)	5′GTA CAA CCC GTA GAT CCG A 3′	56
miR‐101 (3P)	5′GCT GCT ACA GTA CTG TGA T 3′	53.6
miR‐16 (5P)	5′GAC AGT AGC AGC ACG TAA AT 3′	57
Reverse	5′GAG GAA GAA GAC GGA AGA AT 3′	NA
Stem Loop	5′GAAAGAAGGCGAGGAGCAGATCGAGGAA GAAGACGGAAGAATGTGCGCTCGCCTTCTTTC 3′	NA

*Note:* NA: Not available.

**TABLE 3:** Primer sequence of mRNA expression analysis.

Primer name	Primer sequences (5′‐3′)	Annealing temp (°C)
mTOR	F: GCATGAATCGGGATGATCG R: CTGCTGCTGTGTGATTTCTT	56.5
AKT1	F: TCAAGAAGGAAGTCATCGTGGC R: ACAAAGCAGAGGCGGTCGT	64
PI3KCA	F: ACAGCCACACACTACATCAG R: CAGTTGTCCATCGTCTTTCACC	60
ABL	F: TGGAGATAACACTCTAAGCATAACTAAAGGT R: GATGTAGTTGCTTGGGACCCA	60

**TABLE 4:** Demographic characteristics of AML cases and controls.

Parameter assessed	AML cases (*N*, %)	Controls (*N*, %)
(*N* = 21)	(mean ± SD)	(mean ± SD)
Age (year)	50.48 ± 20.33	31.33 ± 2.78
Gender (F/M)	11/10	6/3
Blast %	62.81 ± 20.67 (%)	0
White cell count—× 10^9^/L	25.89 ± 30.49	7.30 ± 2.01
Platelet count—× 10^9^/L	68.62 ± 56.76	365.0 ± 124.7
Red blood cell count—× 10^12^/L	2.77 ± 0.75	4.64 ± 0.17
Sample source (pb/bm)	6/11	9/0
Fab classification (no)		
M0–M1	2	0
M2	5	0
M3	2	0
M4–M5	5	0
None M3	7	0

### 2.5 Statistical Analysis

The mean and standard error of mean (SEM) from two independent experiments were used to express the results. The Student's *t*‐test was used to examine the differences between the two groups. Spearman's correlation analysis was used to ascertain the relationship between the levels of *mTOR*, *AKT*, and *PI3K* mRNAs in AML patients. GraphPad Prism version 9 (GraphPad Software Inc., San Diego, CA, USA) and SPSS version 26 (SPSS Inc., Chicago, IL, USA) were employed for statistical analysis. The 2^−ΔΔCt^ method was used to analyze the relative changes in gene expression from RT‐qPCR experiments. A *p* value of less than 0.05 was considered to indicate a statistically significant difference.

**FIGURE 1:**
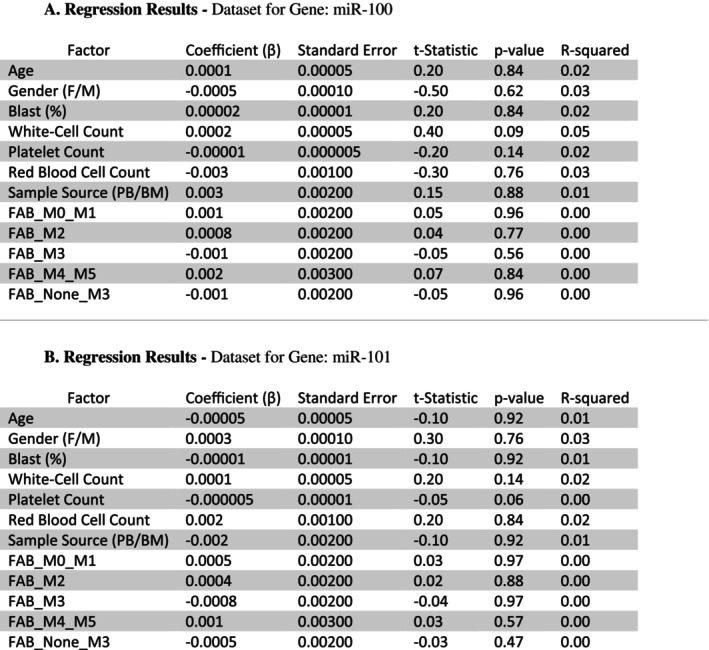
(A) Regression results—dataset for gene: miR‐100. (B) Regression results—dataset for gene: miR‐101.

In the present study, multiple regression analysis was performed to evaluate the expression levels of the *miR‐100* and *miR‐101* genes while accounting for potential moderator variables. This approach allowed for a comprehensive assessment of how these variables influence the relationship between miRNA expression and other key factors. The analysis was conducted using statistical software, ensuring robust and reliable results. This method provides deeper insights into the underlying interactions and the role of miR‐100 and miR‐101 in the context of AML (Figure 1A and Figure 1B).

## 3 Results

Thirty samples were analyzed (21 from AML patients and 9 from the control group, respectively), and their demographic characteristics are shown in Table 4. There was no significant difference in gender distribution between AML patients and the control group (*p* value: 0.49). At the time of diagnosis, the mean leukocyte count was 25.89/μL (ranging from 0.4 μL to 101.0 μL).

### 3.1 Upregulation of miR‐100 Expression

**FIGURE 2:**
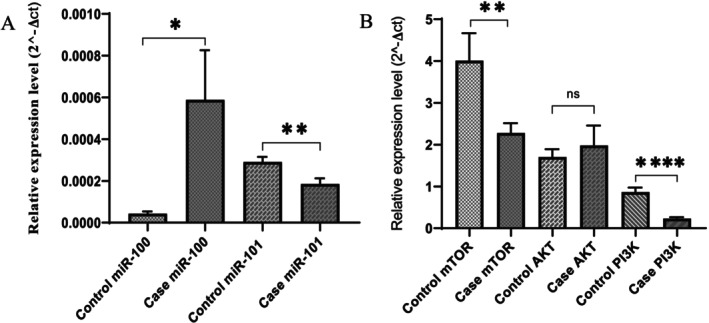
Expression of miR‐100, miR‐101, and mTOR/AKT/PI3K signaling pathway in PB and BM samples from AML patients. (A) Relative expression levels of miR‐100 and miR‐101. The relative expression of miR‐101 decreased, while miR‐100 increased in both PB and BM samples. miR‐16 served as an internal reference. (B) Relative expression levels of mTOR/AKT/PI3K in PB and BM samples of AML patients. The expression levels of mTOR and PI3K decreased in both PB and BM samples. ABL served as an internal reference. Statistical significance is indicated by *, **, and **** for *p* values of < 0.05, < 0.01, and < 0.001, respectively.

The expression of *miR‐100* in bone marrow and peripheral blood specimens from 21 AML patients and 9 healthy individuals (controls) was analyzed using qRT‐PCR. A comparison of relative gene expression of *miR‐100* between healthy controls and patients indicated a 6.8‐fold increase (*p* value: 0.033) (Figure 2A). Furthermore, we also examined the correlation between *miR‐100* expression and features of AML patients. *miR‐100* did not show significant expression changes in FAB classification subtype (M3, Non‐M3) (*p* value: 0.19), sex (*p* value: 0.19), and sample source (*p* value: 0.64).

### 3.2 Downregulation of miR‐101 Expression

A comparison of relative gene expression of *miR‐101* between controls and patients indicated a 0.61‐fold decrease (*p* value: 0.019) (Figure 2A). We also explored the relationship between *miR‐101* and patients' characteristics. Our analysis showed no correlation between *miR‐101* expression level and blast percentage increase (*p* value: 0.09), sex (*p* value: 0.35), PB and BM samples (*p* value: 0.65), and FAB classification subtype (M3, Non‐M3) (*p* value: 0.83).

### 3.3 mTOR: A Key Target of miR‐100 and miR‐101

**FIGURE 3:**
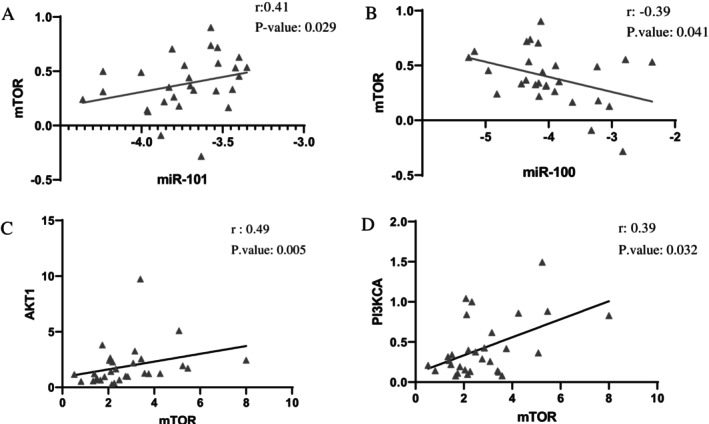
Correlation of mTOR expression with miR‐100, miR‐101, AKT, and PI3K in BM and PB samples from AML patients. (A, B) The correlation between log10(2^−ΔCt^) mTOR expression levels and miR‐100/miR‐101 in BM and PB of AML patients. (C, D) The correlation between mTOR and AKT/PI3K gene expression in BM and PB of AML patients. Spearman's correlation analysis was performed with AML patients and controls (*n* = 30).

To understand the mechanisms underlying the roles of *miR‐100* and *miR‐101*, we estimated their binding sites with the *mTOR* gene using databases such as TargetScan, miRDB, and miRanda. Our findings revealed a significant relationship between *miR‐100* (*r* = −0.39) and *miR‐101* (*r* = 0.41) expression levels with the *mTOR* gene in AML patients (*p* value, respectively: 0.041, 0.029) (Figure 3A,B). No significant correlation was found between *miR‐100* and *miR‐101* expression and the *AKT1* (*p* value, respectively: 0.566, 0.983) and *PI3KCA* (*p* value, respectively: 0.0897, 0.2088).

### 3.4 Expression of mTOR, AKT, and PI3K

We examined the expression of *mTOR*, *AKT*, and *PI3K* in AML patients. Our analysis showed a significant decrease in the mRNA level of these genes in AML cases compared to the control group (Figure 2B). The mean ± SEM of relative gene expression of *mTOR*, *AKT1*, and *PI3KCA* in patients was 2.281 ± 0.23, 1.99 ± 0.47, and 0.23 ± 0.03, respectively, as opposed to 4.01 ± 0.66, 1.70 ± 0.19, and 0.86 ± 0.11 in the normal group. The relative gene expression analysis indicated a 0.56‐, 0.80‐, and 0.25‐fold decrease in *mTOR*, *AKT1*, and *PI3KCA* expression in patients compared to controls, respectively. The *t*‐test revealed a significant decrease in *mTOR* (*p* value: 0.004) and *PI3KCA* (*p* < 0.0001) expression levels in cases compared to controls, while *AKT*1 expression did not differ significantly.

### 3.5 mTOR and PI3K Expression in PB and BM

We evaluated the expression level of *mTOR* and *PI3K* genes between PB and BM samples. The results indicated a significant difference in the expression of *mTOR* (*p* value: 0.025) and *PI3K* (*p* value: 0.015) genes between the two groups based on the source of sample collection. In contrast, no significant difference was detected in the expression of the analyzed genes regarding age, gender, and percentage of blasts.

### 3.6 Regulatory Correlations Among mTOR, AKT1, and PI3KCA

Finally, our study observed a meaningful correlation between the expression of *mTOR* and *AKT1* (*r* = 0.49, *p* value: 0.005) and *PI3KCA* (*r* = 0.39, *p* value: 0.032) genes (Figure 3C,D). However, there was no meaningful relationship between the expression of the *AKT1* gene and *PI3KCA*.

## 4 Discussion

Aberrant expression of miRNAs may cause oncogenesis in several cancers, including AML [16]. In this study, we investigated the expression levels of *miR*‐*100* and *miR*‐*101* and their effect on genes in the *mTOR*/*AKT*/*PI3K* pathway, which perform an essential function in AML pathogenesis.

Several studies have investigated the expression of *miR‐100* in AML. For example, Zhang et al. indicated that the expression of *miR‐100* was increased in AML compared to the control group [1]. This finding was further validated by Sun et al. who studied the expression of this miRNA in pediatric AML [6]. In our study, *miR‐100* also exhibited increased expression in AML (*p* value: 0.033), corroborating previous research findings. Furthermore, Zhang et al. indicated that the expression level of *miR‐100* increased in all subtypes of AML patients, with variations among subtypes [1]. However, our study did not observe differences in *miR‐100* expression among the various subgroups of AML. This discrepancy may be due to our division of AML subgroups into two groups, M3 and non‐M3, due to the lack of sufficient samples of different subtypes.

The *mTOR*/*AKT*/*PI3K* cascade represents a crucial signaling system that is often dysregulated in various human cancers. This pathway has been identified as a key factor in the development of hepatocellular carcinoma and is regulated by several miRNAs, including *miR‐99a*, *miR‐100*, and *miR‐149*, which inhibit *mTOR*, as well as *miR‐9*, *miR‐30*, *miR‐125*, *miR‐149*, and *miR‐379*, which inhibit *AKT* [17, 18]. Research by Ida Rapa et al. demonstrated that the expression of *miR‐100* in tissue negatively regulates *mTOR* mRNA levels. Higher levels of *miR‐100* were associated with more aggressive cancer traits and accelerated disease progression. They showed that regulation of *mTOR* mRNA and TORC1 protein levels by *miR‐100* leads to enhanced cell migration and reduced cell proliferation in H727 and UMC11 lung carcinoid cell lines. Additionally, *miR‐100* affected the sensitivity of these cells to the drug rapamycin [19]. In line with these observations, our study revealed a statistically significant correlation (*p* value: 0.041, *r*: −0.387) between elevated *miR‐100* expression and reduced *mTOR* gene expression; however, no significant correlation was detected with *AKT* or *PI3K*. It appears that the overexpression of *miR‐100* modulates the *mTOR* signaling pathway through direct targeting of *mTOR*, potentially contributing to the progression of AML. Xu et al. also confirmed the link between *miR‐100* and *mTOR* in bladder cancer patients using *miR‐100* inhibitor and mimic, showing that *miR‐100* directly targets *mTOR* [12]. In another study by X‐J Li et al., *miR‐99a* and *miR‐100* were shown to repress expansion and increase apoptosis in acute lymphoblastic leukemia by directly targeting *FKBP51* and *IGF1R*/*mTOR* [20]. Our findings further support this notion, suggesting that *miR‐100* plays a pivotal role in the growth and pathogenesis of AML through its targeting of the *mTOR* gene.

We also examined the expression of *miR‐101*, which is recognized as a tumor suppressor. Our study demonstrated that *miR‐101* is significantly underexpressed in AML patients compared to the control group. Gonzales‐Aloy et al. revealed that *miR‐101* expression levels were decreased in newly diagnosed AML patients compared to the control group, demonstrating the tumor suppressive role of *miR‐101* in *MLL*‐rearranged AML [8]. Lei et al. found that *miR‐101* caused downregulation by targeting the *mTOR*/*AKT*/*PI3K* pathway in liver fibrosis, suggesting that it operates as an antifibrotic mechanism [21]. Similarly, Lin et al. noted a marked decrease in *miR‐101* expression in osteosarcomas, which inversely correlated with *mTOR* expression, confirming *mTOR* as a direct target and showing that *miR‐101* strongly decreased *mTOR* expression at mRNA and protein levels [7]. Conversely, we demonstrated a statistically significant positive correlation between *miR‐101* and *mTOR* in AML patients (*p* value: 0.029, *r*: 0.41), while there was no significant relationship observed between *miR‐101* and *AKT*, *PI3K* genes. This discrepancy may stem from alterations in the expression of other miRNAs, such as *miR‐100*, which also regulates *mTOR* expression. Further contributing factors include genetic variability among patients, cancer‐type–specific expression of *miR‐101* and *mTOR*, environmental factors, variations in the methodologies, and the potential modulation by other regulatory elements or molecules unique to certain cancers.

Hyperactivation of the *mTOR*/*AKT*/*PI3K* signaling pathway is observed in 50%–80% of AML patients [22]. While extensive research has explored the heightened activity of this pathway in AML, there remains a paucity of studies focused on the gene expression within this pathway. This gap underscores the need for additional research to shed more light on this critical area of cancer biology. Hyperactivation of the *mTOR*/*AKT*/*PI3K* pathway was observed alongside increased *mTOR* expression in esophageal carcinoma samples compared to normal tissue. Elevated levels of *mTOR* and related signaling molecules were associated with larger tumor sizes, lymph node metastasis, and more advanced TNM stages. High *mTOR* expression was identified as an independent negative predictor of overall survival rates [23].

*mTOR* expression is elevated in various cancers, including gastric, liver, laryngeal carcinoma, pancreatic, prostate, ovarian, and multiple myeloma [24]. In non–small cell lung cancer (NSCLC) with EGFR mutations, *mTOR* expression levels were low to intermediate in most cases (62.5%), while a significant portion (37.5%) exhibited high *mTOR* expression. Notably, patients with elevated levels of both *mTOR* and *BIM* experienced reduced overall survival and shorter progression‐free survival when treated with erlotinib [25]. Another study revealed a correlation between elevated *mTOR* and phosphorylated *mTOR* (p‐*mTOR*) levels and an increased likelihood of relapse in pediatric acute lymphoblastic leukemia (ALL). Over half of the ALL cases exhibited upregulation of *mTOR*, with chemotherapy‐resistant patients showing a greater average increase in *mTOR* expression [26]. Diverse studies have demonstrated upregulation of various genes within this pathway across different cancers. The *AKT* gene exhibits increased expression in ameloblastoma [27], adenocarcinoma [28], and colon cancer [29]. Ismael Riquelme et al. reported upregulation of all three proteins in gastric cancer [30]. In the present study, we found downregulation in the genes of this pathway, likely influenced by the increased and decreased expression of *miR‐100* and *miR‐101*, respectively.

Our study showed no correlation between the expression of *miR‐100*, *miR‐101*, and *mTOR*, *AKT*, *PI3K* with gender, age, blast percentage, sample source, and AML subtypes. However, there is a significant association between the expression of *mTOR* genes and *AKT* and *PI3K*, as shown by previous studies [31]. These findings suggest that inhibitors of the *mTOR* gene, such as *miR‐100* and *miR‐101*, can affect other genes in this pathway.

The observed downregulation of mTOR and PI3K in our AML cohort presents an intriguing contrast to the widely reported hyperactivation of the mTOR/AKT/PI3K pathway in AML. This discrepancy highlights the complexity and heterogeneity of AML pathophysiology. Several factors could explain these differences, including variations in patient demographics, genetic backgrounds, disease stages, and experimental methodologies. For instance, while many studies focus on mTOR/AKT/PI3K hyperactivation in specific AML subtypes or under certain microenvironmental conditions, our findings may reflect distinct regulatory dynamics influenced by miR‐100 and miR‐101 expression. Additionally, differences in sample preparation, analytical techniques, and cohort size might contribute to variability in pathway activity measurements. To reconcile these findings, further research should explore the interplay between miR‐100/miR‐101 and the mTOR/AKT/PI3K axis in diverse AML contexts, integrating both functional studies and broader clinical datasets. This approach could help clarify the role of these pathways in AML progression and their potential as therapeutic targets.

The present study had limitations, such as sample size and bone marrow sampling and storage. Despite these limitations, the results indicate that additional research is required to confirm the function of *miR‐100* and *miR‐101* and their direct targets in AML to control disease progression and prevent disease recurrence.

The inclusion of APL M3 patients in our study was deliberate to capture the diverse landscape of AML and provide a holistic analysis of miR‐100 and miR‐101 involvement across different subtypes. While it is true that APL M3 is distinct in terms of treatment protocols and prognosis, this subtype shares underlying leukemogenic mechanisms that can intersect with other AML subtypes, particularly in pathways like mTOR signaling. By analyzing miR‐100 and miR‐101 expression in APL M3 alongside other AML subtypes, we identified potential shared and unique regulatory patterns that contribute to AML heterogeneity. We acknowledge that this approach introduces variability, which we have addressed by carefully interpreting findings specific to APL M3 patients. This inclusion underscores the complexity of AML and supports the need for further subtype‐specific investigations to refine the therapeutic implications of miR‐100 and miR‐101 targeting, particularly in the context of mTOR pathway engagement.

The correlation analysis in our study provides valuable insights into the potential roles of miR‐100 and miR‐101 in the mTOR/AKT/PI3K signaling pathway in AML; however, we acknowledge its limitations in establishing causality. The lack of significant correlation between miR‐100/miR‐101 and AKT expression highlights the complexity of miRNA‐mediated regulatory networks and suggests that additional layers of control might influence pathway dynamics. Interestingly, our observation of mTOR and PI3K downregulation contrasts with reports of mTOR/AKT/PI3K hyperactivation in AML, reflecting the heterogeneity of AML subtypes and patient‐specific molecular profiles. This discrepancy underscores the need for further experimental validation, such as overexpression or knockdown studies, to confirm the functional role of these miRNAs and their downstream effects. These experiments, along with broader cohort analyses, would help reconcile conflicting findings in the literature and clarify how miR‐100 and miR‐101 modulate the mTOR/AKT/PI3K axis in different AML contexts. By addressing these gaps, future research can build on our study to deepen the understanding of miRNA‐driven mechanisms in AML pathogenesis.

## 5 Conclusion

Despite studies and research, the results of long‐term remission of AML patients are disappointing. As a result, our study found that *miR‐101* and *miR‐100* are tumor suppressor genes that are frequently downregulated and upregulated, respectively, in AML patients. Dysregulation of *miR‐100* and *miR‐101* is associated with reduced expression of *mTOR*. Accordingly, *miR‐100*, *miR‐101*, and the *mTOR* pathway are potential therapeutic targets for AML.

## Author Contributions

M.K. conducted experiments, analyzed and interpreted the data, drafted the manuscript, and revised the manuscript. M.H.M. and M.A.F. drafted the review and directed the work. M.R. and S.S.B. reviewed and revised the manuscript. J.G. and M.G. analyzed the data. All authors have read and approved the final manuscript for publication.

## Ethics Statement

All procedures performed in studies involving human participants were in accordance with the ethical standards of the institutional and/or national research committee and with the 1964 Helsinki declaration and its later amendments or comparable ethical standards. This study received confirmation from Shahid Beheshti University of Medical Sciences Ethics Committee (Ethics ID: IR.SBMU.RETECH.REC.1400.693), and it was carried out by the rules established by Taleghani Hospital Ethics Committee, which is a member hospital of Shahid Beheshti University of Medical Sciences, and written consent was obtained from the patients or their guardians.

## Consent

The authors have nothing to report.

## Conflicts of Interest

The authors declare no conflicts of interest.

## Supporting information


**Supplementary Figure 1** Melting curves A, B, C, D, E, and F represent *ABL*, *PI3KCA*, *AKT1* and the *mTOR* gene, miR‐100, and miR‐101, respectively. A particular, singular peak signifies the primers’ specificity and a lack of contamination.

## Data Availability

The datasets used or analyzed during the current study, as well as the study protocol, are available from the corresponding author on reasonable request.
