# Supplementary material for: Targeting the mTOR Signaling Pathway Through miR‐100 and miR‐101 in De Novo Acute Myeloid Leukemia: Implications for Therapeutic Intervention
Source: Cancer Rep (Hoboken). 2025 Aug 3;8(8):e70264. doi: 10.1002/cnr2.70264 (PMC12318680; doi:10.1002/cnr2.70264)

**Supplementary Fig. 1.** Supplementary Figure 1 Melting curves A, B, C, D, E and F represent ABL, PI3KCA, AKT1 and the mTOR gene, miR-100, and miR-101 respectively. A particular, singular peak signifies the primers' specificity and a lack of contamination.

**Supplementary Fig. 1**A: Melting curve ABL


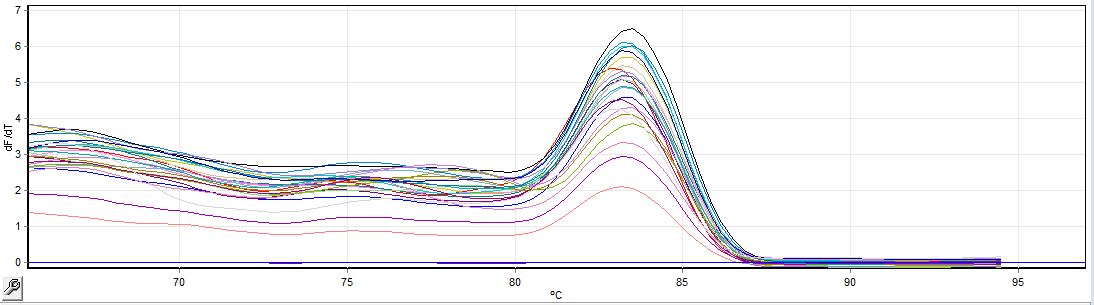


**Supplementary Fig. 1**B: Melting curve PI3KCA


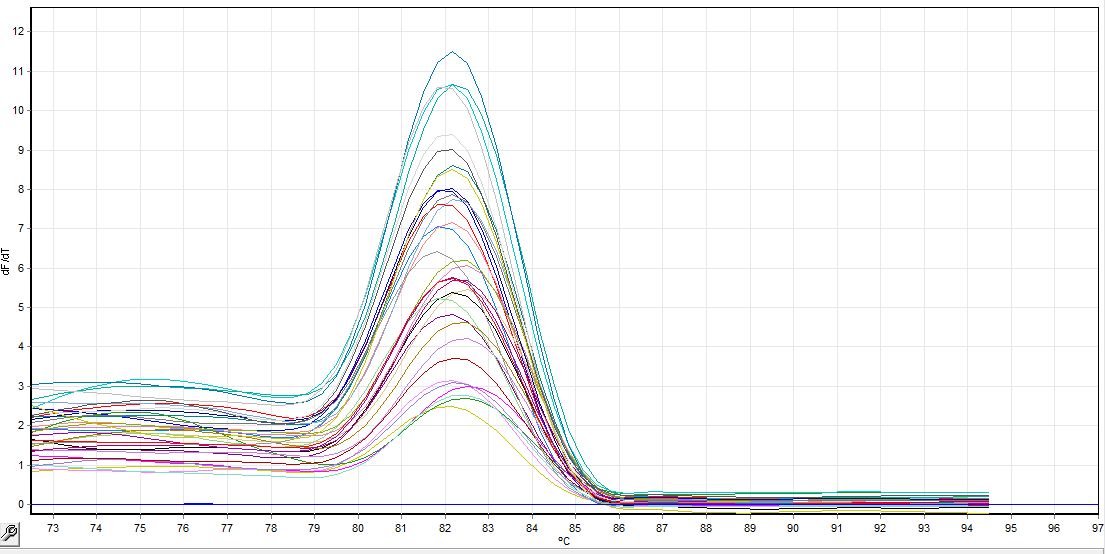


**Supplementary Fig. 1**C: Melting curve AKT1


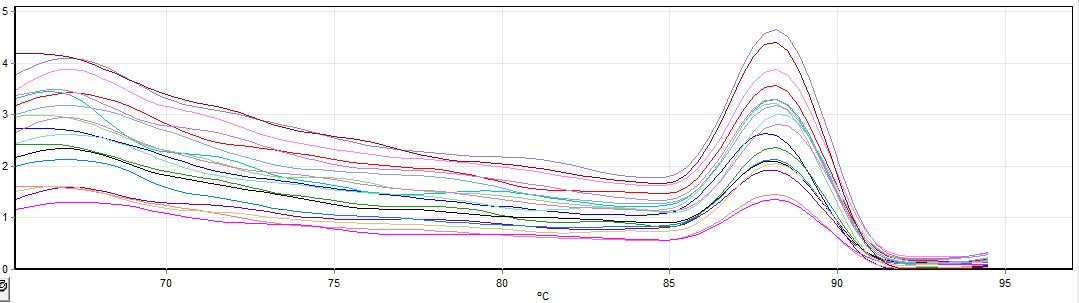


**Supplementary Fig. 1**D: Melting curve mTOR


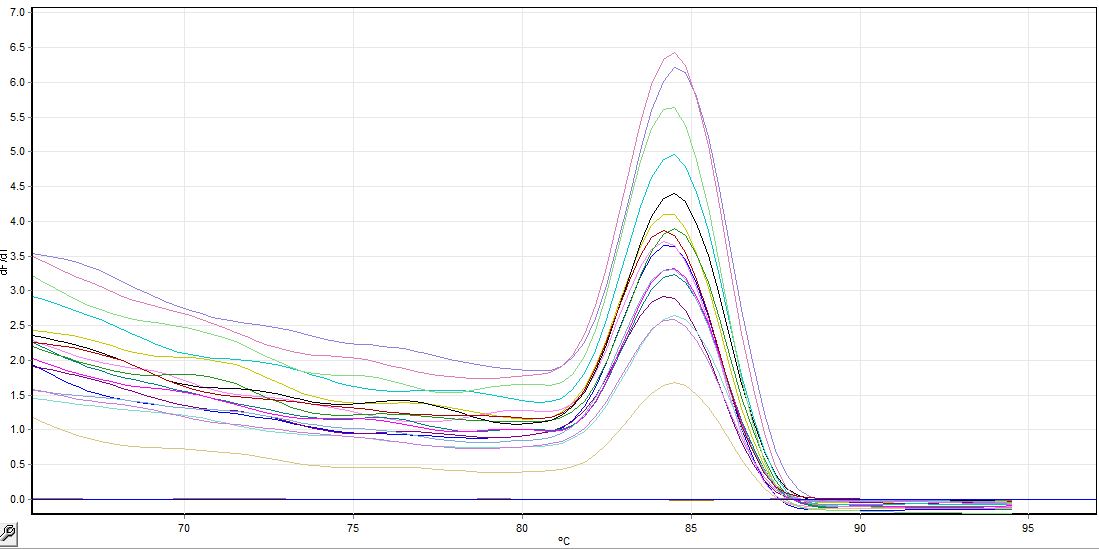


**Supplementary Fig. 1**E: Melting curve miR-100


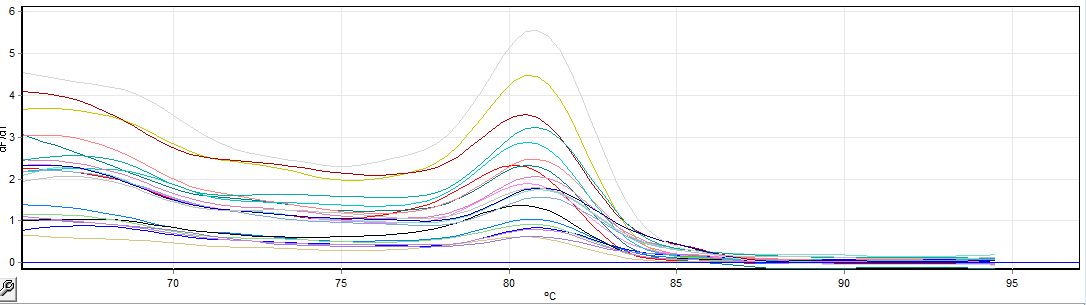


**Supplementary Fig. 1**F: Melting curve miR-101


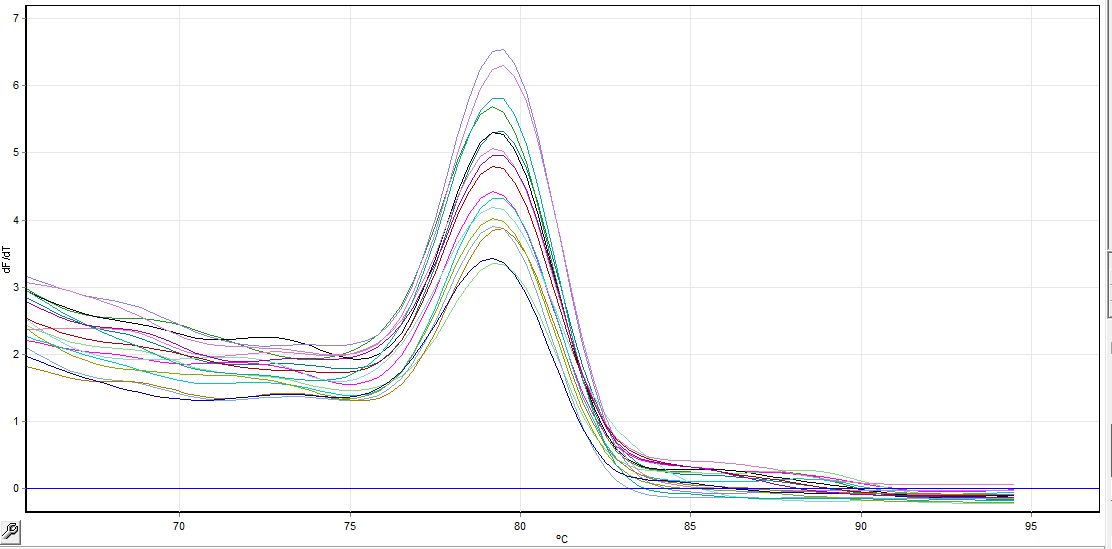

Supplement: Supplementary file 1 — Supplementary Figure 1 Melting curves A, B, C, D, E, and F represent ABL, PI3KCA, AKT1 and the mTOR gene, miR‐100, and miR‐101, respectively. A particular, singular peak signifies the primers’ specificity and a lack of contamination. [file CNR2-8-e70264-s001.docx]
